# Contextual and personal factors as predictors of interpersonal conflict in emerging adult men

**DOI:** 10.3389/fpubh.2026.1778422

**Published:** 2026-04-22

**Authors:** Ryan Rullo, David T. Lardier, Daniel Kim, Keosha T. Bond, Trace Kershaw, S. Raquel Ramos

**Affiliations:** 1 School of Nursing, Yale University, Orange, CT, United States; 2 Department of Psychiatry and Behavioral Sciences, Division of Community Behavioral Health, The University of New Mexico Health Sciences Center, Albuquerque, NM, United States; 3 School of Community Health and Behavioral Sciences, Bouvé College of Health Sciences, Northeastern University, Boston, MN, United States; 4 School of Medicine, Community Health and Social Medicine, City University New York, New York, NY, United States; 5 Department of Social and Behavioral Sciences, School of Public Health, Yale University, New Haven, CT, United States; 6 Center for Interdisciplinary Research on AIDS, New Haven, CT, United States

**Keywords:** conflict, emerging adult, health disparities, housing, socioeconomic status

## Abstract

**Introduction:**

Social determinants are contextual factors that influence health outcomes through individuals’ living, working, and social conditions, alongside overarching structural dimensions that shape these conditions. In contrast, demographic factors refer to personal characteristics, such as age and ethnicity, which shape individual experiences. Understanding these experiences requires examining how aspects of identity interact with one another and with societal structures of power. The purpose of this study was to examine the associations between contextual and personal factors and interpersonal conflict among emerging adult men.

**Methods:**

A secondary analysis of 322 individuals utilized multiple linear regression analyses to assess the relationships between contextual factors and interpersonal conflict experiences. Key predictors included age, race/ethnicity, education status, employment status, individual income, and current health insurance. Moderation analyses were performed to explore the interactive effects of these individual characteristics and perceptions of physical safety on interpersonal conflict.

**Results:**

Our findings revealed several significant predictors of interpersonal conflict. Notably, lack of health insurance was positively associated with experiences of interpersonal conflict (*β* = 0.09, *p* < 0.001). Unemployment (*β* = 0.58, *p* < 0.01) and housing instability (*β* = 0.65, *p* < 0.001) also significantly contributed to this outcome. Concerns about safety in living environments correlated with increased experiences of interpersonal conflict (*β* = 0.58, *p* < 0.001). Additionally, age positively influenced interpersonal conflict among Hispanic participants (*β* = 0.05, *p* < 0.01), indicating that older individuals reported heightened levels of conflict. Interaction effects demonstrated that Hispanic participants with greater concerns about physical safety experienced increased levels of interpersonal conflict (*β* = 0.72, *p* < 0.05).

**Discussion:**

This study identified that external factors, such as lack of health insurance, unemployment, housing instability, and safety concerns were associated with experiences of interpersonal conflict. Unmet basic needs, like housing, can lead to transactional behaviors, which elevate health risks. Future studies should employ mixed methods and longitudinal designs to explore the mechanisms underlying these associations. Implications for practice include healthcare providers incorporating routine assessments of housing stability and safety concerns into clinical encounters. Implications for policy include prioritization for increasing access to healthcare and addressing structural barriers.

## 1 Introduction

Social determinants are contextual factors influencing health outcomes through individuals’ living, working, and social conditions, as well as the overarching structural and social systems that shape these conditions (1). Key determinants include employment status, education, income, housing, perceived physical safety, and health insurance coverage. In contrast, sociodemographic factors refer to personal characteristics such as age, race, ethnicity, which shape individual experiences. Understanding these experiences requires examining how different aspects of identity, such as race, ethnicity, and sexual orientation, interact with one another and with societal structures of power. This concept, known as intersectionality, highlights the unique experiences of interpersonal conflict and privilege that arise from the interplay of multiple identities (2–4).

Underrepresented populations, including men from sexual minority backgrounds often experience challenges due to intersecting stigmas associated with overlapping identities. These stigmas impressed upon individuals contribute to economic instability, workplace bias, housing insecurity, and increased risks for violence and interpersonal conflict (5–8). Structural factors often limit access to healthcare, resulting in delayed or inadequate care for underrepresented groups, which exacerbates poor health outcomes (9, 10). High unemployment rates and financial instability further perpetuate experiences of interpersonal conflict, contributing to housing instability and increasing the risk of homelessness among men from sexual minority backgrounds (9, 11, 12). Such instability is linked to heightened mental health challenges and substance use (13). The compounded effects of interpersonal conflict experienced by individuals with multifaceted identities can lead to interpersonal biases (14, 15). For instance, data indicate that ethnic and racial minority individuals and those with lower incomes report lower perceived neighborhood safety and housing stability compared to their non-minority counterparts (16). These challenges are further exacerbated by family rejection, language barriers, and cultural stigmas (17–19).

Given these challenges, housing and other contextual factors play central roles in shaping health outcomes and promoting health optimization. For example, the risk of HIV in men from sexual minority backgrounds is heightened due to interrelated factors, which can lead to increased housing instability and economic challenges. For individuals living with HIV, the experience of interpersonal conflict often exacerbates mental health issues, contributing to a higher risk of suicide (20). Additionally, this contributes to missed medical appointments and decreased medication adherence, which are frequently linked to a lack of trust in healthcare providers (21, 22). The compounded effects of interpersonal conflict and stigma can lead to poor sleep, financial instability, and increased cardiovascular risk (23). Furthermore, stigma and interpersonal conflict can result in negative internalized feelings, concealment of romantic relationships, transactional relationships, and reduced knowledge of HIV prevention strategies (10, 24). Ultimately, no individual should be subjected to such adverse conditions and challenges as everyone deserves the opportunity to live a fulfilling and healthy life, free from stigma and instability.

While prior studies have identified associations between contextual factors and interpersonal conflict more broadly, the present study specifically addresses gaps related to ethnic and racial emerging adult men from sexual minority backgrounds. This study examines the intersections of age, physical safety, and identity, which remain insufficiently explored in existing literature. To effectively guide future interventions, more information is needed on how intersecting identities and experiences of interpersonal conflict impact social determinants. The purpose of this study is to examine the associations between contextual factors, personal characteristics and experiences of interpersonal conflict in a sample of 322 emerging adult men from sexual minority backgrounds.

Our research questions are as follows:

Research Question 1: What are the relationships between contextual factors and interpersonal conflict experiences?

Research Question 2: How do individual-level personal characteristics influence interpersonal conflict experiences?

Research Question 3: Is Hispanic ethnicity a potential moderator in the relationship between contextual factors and interpersonal conflict experiences?

## 2 Theoretical framework

We used a comprehensive social-ecological approach to understanding and addressing health disparities. This framework identifies a range of determinants contributing to disparities and highlights five key domains of influence, (1) biological, (2) behavioral, (3) physical/built environment, (4) sociocultural environment, and (5) healthcare system factors. Each domain is examined across levels of influence including individual, interpersonal, community, and societal (25). While the individual and interpersonal levels are distinct, they are often analyzed together as structural factors due to their collective impact on systemic health inequities and are included within the sociocultural environment domain. This multi-layered approach facilitates the identification of complex and interconnected factors affecting health outcomes, particularly among minority and underrepresented populations. The framework emphasizes the importance of integrating varied scientific methods to comprehensively assess how contextual factors, healthcare access, biological processes, and environmental factors contribute to health disparities. This integration guides researchers in developing targeted and effective interventions (25).

The sociodemographic factors collected in this study correspond to the individual factors described in the model (25), including overlapping identities (such as ethnicity, ability, and age), employment status, socioeconomic status, and insurance coverage. Experiences of interpersonal conflict examined in this study, such as negative service encounters, negative preconceptions, and threats or harassment, which relate to the interpersonal and societal factors within the model. Although this study does not directly measure the societal factors, these factors significantly impact many of our study variables, including physical safety, housing and employment access, and educational status.

## 3 Methods

### 3.1 Design

The primary study, from which the data is derived, utilized a mixed-methods embedded design within a randomized controlled trial (RCT) (26). Initially, qualitative methods were employed to design an HIV self-testing infographic, with a leadership team of HIV experts conducting focus groups and discussions to ensure the infographic was user-friendly and informative. Following this design phase, we implemented an online RCT to assess the feasibility, acceptability, and comprehension of the infographic among emerging adult men from sexual minority backgrounds. In addition to evaluating the infographic, we collected data on social determinants to understand how these factors might influence participants’ experiences and perceptions related to HIV self-testing. We utilized a national recruitment panel to ensure those meeting eligibility were enrolled. Details of the primary study are provided elsewhere (26). The present secondary analysis examined the association of contextual factors, personal characteristics on intersectional conflict among emerging adult men from sexual minority backgrounds. The primary study received approval from the institutional review boards at Yale University (#1610018552) and New York University (IRB-FY2018-1573).

### 3.2 Participants

From 2017 to 2019, a total of 322 men from sexual minority backgrounds, ages 18 to 34 years, participated in the study (26). Inclusion criteria were: (1) ages 18–34, (2) HIV serostatus negative or status unknown, (3) male sex, (4) able to understand English. The primary aim of the study was to develop and assess the feasibility, acceptability, and comprehension of the HIV self-testing infographic. Participants were recruited from across the United States, including Puerto Rico, through an online national research recruitment service. All participant data were de-identified prior to review and analysis.

### 3.3 Measures

#### 3.3.1 Predictors

Several sociodemographic and contextual factor variables were included in the analysis. These variables encompassed age (in years), race/ethnicity, education status, employment status, individual income, and current health insurance.

- Age was measured in years (*M* = 26.35, SD = 4.66, range = 18–34).
- Race/ethnicity was coded as a series of dichotomous variables (yes = 1, no = 0) for categories including Hispanic, Black/African American, Asian/Pacific Islander, Arab/Middle Eastern, American Indian/Native American, and White.
- Education was categorized using seven items, ranging from less than high school (1) to professional degree (5).
- Individual income was assigned to seven categories, from less than $10,000 per year (1) to more than $150,000 per year (6).
- Current health insurance was measured using seven items, including uninsured (1), private health insurance (2), parent’s health insurance (3), state-sponsored health insurance (4), Medicaid (5), military health care (6), and no health insurance/uninsured (7).
- Current living situation was categorized using five items, ranging from living independently (1) to living on the street (4) or with a person in exchange for sex (5).
- Physical safety in the living location was assessed dichotomously (0 = Yes, I feel physically safe, 1 = No, I do not feel physically safe).

All sociodemographic and contextual variables were analyzed in accordance with their original survey response formats. Variables with fixed, non-interval response options (e.g., race/ethnicity, education, income, employment, health insurance, living situation) were treated as categorical to avoid imposing inappropriate ordinal structure. Redundant health insurance categories reflecting lack of coverage were collapsed into a single ‘uninsured’ category. Physical safety was modeled dichotomously as it was collected through a binary question.

#### 3.3.2 Outcome

The primary outcome was interpersonal conflict. This was measured using The Everyday Discrimination Scale, a nine-item measure assessing day-to-day experiences of interpersonal conflict, was utilized to evaluate interpersonal conflict experiences (e.g., “You are treated with less courtesy than other people”; “People act as if they are afraid of you”; “You are threatened or harassed”) (27, 28). Responses were collected on a 5-point Likert scale ranging from never (0) to almost every day (5). Prior studies have demonstrated high reliability (29, 30) and construct validity (31–33) for this scale. Although some studies have indicated performance differences among Hispanic/Latine and Asian American responses (34), most psychometric studies emphasize the scale’s performance across racial/ethnic groups (34–37). In the current sample, responses summed across the nine items ranged from 0 to 45, with a mean of 43.50 (SD = 13.37; *α* = 0.90; McDonald’s *Ω* = 0.96).

The Everyday Discrimination Scale was selected to capture perceptions relevant to interpersonal conflict, recognizing that experiences of discrimination often manifest in relational dynamics. While the scale primarily assesses perceived discrimination, we argue that it serves as a valuable proxy for understanding interpersonal conflict among ethnic and racial men from sexual minority backgrounds. Our focus encompasses both experiences of discrimination and the broader spectrum of interpersonal conflict.

### 3.4 Statistical analysis

All analyses were conducted using R, version 4.4.1. Preliminary analyses included assessments of normality, descriptive statistics, and reliability measures (Cronbach’s alpha and McDonald’s omega). Among the 322 completed surveys, less than 5% of the data were missing. To handle missing data, multiple imputation by chained equations (MICE) was employed (38), utilizing 20 imputations and 50 iterations to preserve statistical power and precision. Variable-appropriate methods (e.g., ordinal, binary, continuous) were applied during imputation, ensuring the maintenance of underlying relationships among variables and enhancing the robustness of statistical results. Diagnostics were conducted to evaluate the imputation process, with no major concerns identified when comparing strip plots of imputed versus observed values.

Multiple linear regression analyses were conducted to examine the associations between contextual factors, personal characteristics, and interpersonal conflict. Moderation analyses explored the interacting effects of Hispanic identity and several personal characteristics on interpersonal conflict. Moderation analyses also examined the interacting effects of Hispanic identity and physical safety on interpersonal conflict.

Assumption testing was completed to assess for heteroscedasticity and multicollinearity. Linearity was not evaluated, as all primary predictors were categorical and therefore not subject to linearity assumptions. Homoscedasticity was assessed using the studentized Breusch–Pagan test (45, 46). Multicollinearity was evaluated using variance inflation factors (VIF), with generalized VIFs adjusted for factor degrees of freedom for multi-level categorical predictors. Assumption testing indicated no violation of homoscedasticity. The studentized Breusch–Pagan test was nonsignificant, χ^2^(32) = 27.61, *p* = 0.69, providing no evidence of heteroscedasticity in the residuals. Multicollinearity diagnostics showed that all predictors had acceptable levels of shared variance; generalized variance inflation factors (GVIFs) and adjusted GVIF values were low (GVIF_adj_ range = 1.05–1.57), well below commonly used thresholds of five. These results indicate that the regression estimates were not adversely affected by heteroscedasticity or multicollinearity. As a robustness check, HC1 heteroscedasticity-consistent standard errors were estimated to account for potential violations of homoscedasticity. Because the substantive inferences remained unchanged, these robust standard errors were retained for all final analyses.

### 3.5 Results

#### 3.5.1 Descriptive statistics

Participants ranged in age from 18 to 34 years, with a mean age of 26.35 years (SD = 4.66) (Table 1). The majority of participants (65.0%) were between the ages of 25 and 34 years. All participants self-identified as male. The racial/ethnic composition revealed that most participants identified as Hispanic (49.0%) or Black/African American (38.2%). Among those identifying as “White” (22.6%), a significant number also reported Hispanic ethnicity or multiple racial/ethnic identities.

**Table 1:** Contextual and Personal Characteristics.

Categorical variables	Total Sample (*N* = 322)
Gender	
Male	322 (100%)
Race-Ethnicity^a^	
Hispanic	157 (48.9%)
Black/African American	122 (38.2%)
Asian/Pacific Islander	69 (21.6%)
Arab/Middle Eastern	10 (3.1%)
American Indian/Native American	17 (5.6%)
White	74 (22.6%)
Multi-racial	225 (69.9%)
US born	
Yes	280 (87.8%)
No	42 (12.2%)
Education	
Less than high school	5 (1.6%)
High school graduate/GED	77 (23.8%)
Some college	75 (23.2%)
2- year degree	38 (12.2%)
4-year degree	95 (29.2%)
Professional degree	32 (10.1%)
Employment status	
Employed full-time	167 (52.0%)
Employed part-time	54 (16.9%)
Self-employed	19 (5.95%)
Unemployed looking for work	30 (9.1%)
Unemployed not looking for work	4 (1.3%)
Student	45 (13.8%)
Disabled	3 (0.9%)
Income	
Less than $10,000	59 (18.3%)
$10,000 - $29,999	84 (26.1%)
$30,000 - $49,999	69 (21.4%)
$50,000 - $69,999	44 (13.7%)
$70,000 - $89,999	29 (9.0%)
$90,000 - $149,000	26 (8.1%)
More than $150,000	11 (3.4%)
Living situation	
In my own place (e.g., apartment)	350 (54.4%)
With family	236 (36.7%)
With friends	74 (11.5%)
Unhoused	10 (1.6%)
With a person that I have exchanged sex	8 (1.2%)
Health insurance	
Private health insurance	117 (36.4%)
Parent’s health insurance	51 (16.0%)
State-sponsored health plan	35 (10.7%)
Medicaid	43 (13.%)
Military health care (TRICARE/VA/CHAMP – VA)	10 (2.8%)
Uninsured	66 (20.7%)
Physical safety in living location	
Yes, I feel physically safe	278 (86.3)
No, I do not feel physically safe	44 (13.7)

Continuous variables	M (SD)
Age in years	26.35 (4.66)
Interpersonal conflict	43.5 (13.37)

^a^Separate responses were recorded for race-ethnicity.

Educational attainment varied within the sample: 1.6% of participants had less than a high school education, while 39% had completed either a four-year college degree (29.2%) or a professional degree (10.1%). Employment status indicated that over half of the participants (52.4%) were employed full-time, and 57% reported an annual income ranging from USD 10,000 to USD 49,999. Additionally, 18% of participants earned less than USD 10,000 annually, while 3.4% reported incomes exceeding USD 150,000 per year.

Regarding living situations, the majority of participants reported residing in their own place, such as an apartment (54.4%), while 36.7% lived with family. Smaller proportions lived with friends (11.5%), on the street (1.6%), or with a person with whom they had exchanged sex (1.2%). In terms of health insurance coverage, over 50% of participants were either on their parent’s health insurance (16.0%) or had private insurance (36.4%), while 20.7% reported having no insurance. Concerning physical safety, 86.3% of respondents indicated feeling physically safe in their living environment, whereas 13.7% reported feeling unsafe. Table 1 outlines means and standard deviations for continuous variables.

#### 3.5.2 Regression analyses

Multiple linear regression analyses for Model 1 identified several significant positive predictors of interpersonal conflict (Table 2). Regression coefficients were estimated using heteroscedasticity-robust HC1 standard errors to correct for non-constant error variance. Predictors reported were statistically significant at *p* < 0.05. Notably, lack of health insurance (*β* = 0.09, *p* < 0.001), unemployment (*β* = 0.58, *p* < 0.01), and housing instability (*β* = 0.65, *p* < 0.001) were all positively associated with experiences of interpersonal conflict. Additionally, lack of physical safety was positively correlated with interpersonal conflict (*β* = 0.58, *p* < 0.001).

**Table 2:** Associations between contextual and personal characteristics with interpersonal conflict using multiple linear regression (*N* = 322).

Predictor variable	Estimate	Robust Std. error	*t*-value	Pr (>|t|)
(Intercept)	13.130	3.515	3.700	**0.000**
Ethnicity^1^				
Hispanic	0.168	0.107	1.671	0.096
Race^1^				
Black/African American	−0.034	0.116	−0.294	0.769
American Indian/Native American	0.213	0.135	1.527	0.218
Asian	0.496	0.256	1.660	0.090
Native Hawaiian or Pacific Islander	0.143	0.219	0.585	0.558
White	0.229	0.123	1.770	0.077
Age^2^	0.038	0.027	2.846	**0.005**
Education status^1^				
High school diploma/GED	−0.108	0.429	−0.253	0.801
Some college	−0.167	0.432	−0.386	0.700
2-year degree	−0.193	0.440	−0.439	0.661
4-year degree	0.075	0.437	0.172	0.864
Professional degree	0.206	0.462	0.445	0.656
Health insurance^1^				
Uninsured	0.094	0.027	3.447	**0.001**
Employment^1^				
Employed part-time	0.059	0.156	0.382	0.709
Self-employed	0.009	0.250	0.037	0.971
Unemployed looking for work	0.575	0.197	2.918	**0.003**
Unemployed not looking for work	0.596	0.502	1.185	0.237
Student	0.087	0.166	0.614	0.608
Disabled	−0.088	0.673	−0.157	0.878
Living situation^1^				
In my own place (e.g., apartment)	−0.027	0.137	−0.195	0.846
With Friends	−0.272	0.166	−1.642	0.102
With Family	−0.187	0.137	−1.364	0.173
Unhoused	0.651	0.146	4.457	**0.000**
Physical safety^1^				
No, I do not feel physically safe	0.577	0.153	3.790	**0.000**
Hispanic identity interaction effects				
Hispanic identity * Age	0.052	0.026	2.033	**0.043**
Hispanic Identity * No, I do not feel physically safe	0.721	0.409	2.250	**0.025**
Model fit indices				
Log likelihood ratio	−409.32			
AIC	886.63			
BIC	1014.97			
Residual deviance	239.61			
Null deviance	321.00			
DF residual	289			
DF null	321			
R^2^	0.254			
Adjusted R^2^	0.171			
N	322			

^1^Predictors are dichotomous (0 = No, not identified group and 1 = Yes, identified group); ^2^Age measured as continuous variable. Std. error, standard error.

Bolded p-values indicate statistical significant (*p* < 0.05).

Interaction effects revealed that age was positively associated with interpersonal conflict among Hispanic participants (*β* = 0.05, *p* < 0.01), indicating that older Hispanic participants experienced higher levels of interpersonal conflict. Furthermore, Hispanic participants who reported greater concerns regarding physical safety also indicated increased levels of interpersonal conflict (*β* = 0.72, *p* < 0.05). No other significant interaction effects were identified. The model fit was deemed acceptable. The model explained approximately 25% of the variance in interpersonal conflict (R^2^ = 0.25, Adjusted R^2^ = 0.17), indicating a modest but meaningful overall fit.

**Figure 1:**
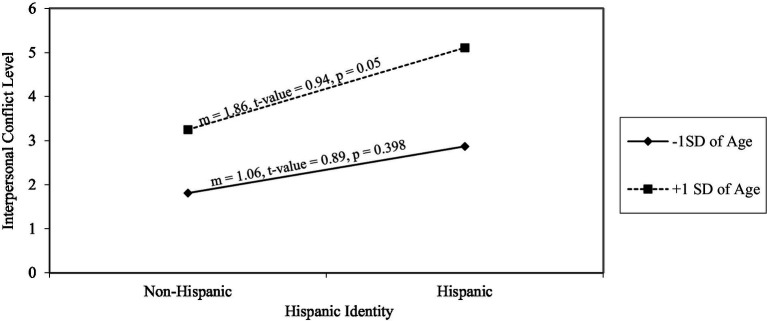
Interaction effects of Hispanic identity and age on interpersonal conflict (*N* = 322). *m*, gradient of slope.

**Figure 2:**
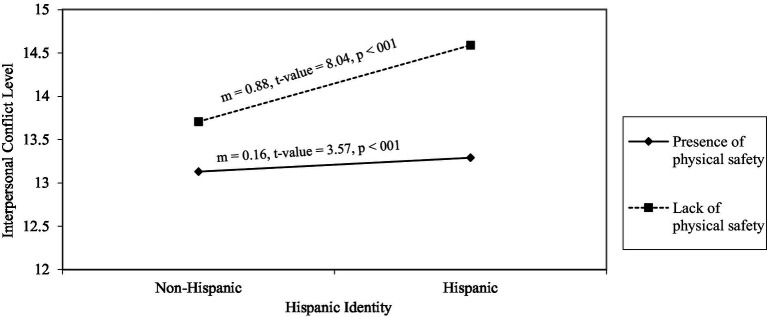
Interaction effects of Hispanic identity and lack of physical safety on interpersonal conflict (*N* = 322). *m* = 0.16, *t*-value = 3.57, *p* < 001. *m* = 0.88, *t*-value = 8.04, *p* < 0.001. *m*, gradient of slope.

Figures 1, 2 provide visual representations of these findings. As illustrated in Figure 1, the simple slope for the interaction between Hispanic identity and age on interpersonal conflict was significant for participants one standard deviation above the mean age (*m* = 1.86, *t* = 0.94, *p* = 0.05). Conversely, the slope was not significant for participants one standard deviation below the mean age (*m* = 1.06, *t* = 0.89, *p* = 0.39).

Figure 2 depicts the interaction effects of Hispanic identity and lack of physical safety on interpersonal conflict. The simple slope for the interaction between Hispanic identity and lack of physical safety was significant for both the presence of physical safety (*m* = 0.16, *t* = 3.57, *p* < 0.001) and the absence of physical safety (*m* = 0.88, *t* = 8.04, *p* < 0.001).

## 4 Discussion

This study identified that external factors, such as lack of health insurance, unemployment, and housing instability were associated with experiences of interpersonal conflict in 322 emerging adult men from sexual minority backgrounds. Our outcome variable, the Everyday Discrimination Scale, served as a pragmatic proxy for interpersonal conflict. The Everyday Discrimination Scale measured routine perceived mistreatment rather than discrete conflict events, but its items captured relational tensions that overlap conceptually with interpersonal conflict that were examined in this study.

Older Hispanic participants were significantly more likely to experience interpersonal conflict, signifying that age may heighten the impact of these contextual factors. We examined Hispanic identity as a potential moderator in line with the socioecological framework, which views sociocultural position as modifying exposure and response to structural stressors. Cultural norms, language and immigration-related barriers, socioeconomic positioning, and distinct patterns of stigma and discrimination may alter how housing instability, unemployment, and lack of insurance translate into interpersonal conflict. Familism could buffer some stressors while also intensifying relational tensions, and limited access to culturally appropriate services or heightened anti-Latino discrimination could amplify conflict and reduce help-seeking. This result is presented as exploratory and requires replication with prespecified hypotheses, larger samples, and appropriate adjustment for multiple comparisons. Moreover, the implications of these findings are profound, particularly in understanding how housing instability and workforce status can lead individuals to seek transactional relationships as a means of survival (10). This can increase risks for sexually transmitted infections, as individuals prioritize basic, fundamental needs like housing, over safer sexual practices. Moreover, physical safety concerns may stem from the dynamics of these transactional relationships, exacerbating experiences of interpersonal conflict. Evidence suggests that individuals facing economic pressures are more likely to engage in risky sexual behaviors, which emphasizes the urgency of addressing these contextual factors in discussions of health disparities (10, 24).

The socioecological model utilized in this study provided a valuable lens for understanding how intersecting identities and contextual factors exacerbate health disparities (25). By employing this framework, we were able to effectively identify the complex role of contextual factors in shaping experiences of interpersonal conflict among men from sexual minority backgrounds. Our findings align with previous research that emphasized the role of contextual factors, such as housing instability and safety concerns, in shaping experiences of interpersonal conflict among underrepresented populations (15, 38). A scientific statement focused on the health and wellbeing of sexual minority populations found that psychosocial stressors, including interpersonal conflict, are influential drivers of health disparities, which are exacerbated by structural inequities that lead to adverse health outcomes (39). Other research has suggested that structural violence, including housing insecurity and barriers to healthcare, amplifies health disparities (40). These findings on structural inequities align with our own results, indicating that housing instability and safety concerns are critical predictors of interpersonal conflict. Evidence from a systematic review found that systemic barriers, such as lack of cultural competence and inequities in healthcare access, worsened interpersonal conflict and health outcomes for individuals with multiple intersecting identities (12). Other studies have also examined structural challenges, highlighting how interpersonal conflict and stigma shape sexual health behaviors (41, 42). This is particularly relevant given the interrelatedness of race, ethnicity, and relationship and intimacy factors within our study population.

Furthermore, research indicates a connection between unsafe neighborhoods and community stigma, which adversely affects mental health and reduces healthcare engagement in persons with HIV (43). This finding resonates with our results regarding the significant role of physical safety in interpersonal conflict experiences, highlighting the broader implications of contextual factors across different racial and ethnic groups and highlights how overlapping identities magnify vulnerabilities. Consistent with prior studies, our findings emphasized that structural inequities, particularly the lack of health insurance and unsafe environments, compound experiences of interpersonal conflict among men from sexual minority backgrounds.

The research framework was crucial in linking the key drivers that systematically disadvantage populations through intersecting societal, environmental, and individual-level mechanisms. These findings collectively reinforce the multifaceted nature of interpersonal conflict and provide a robust foundation for the development of targeted interventions and systemic reforms aimed at addressing contextual factors and improving health optimization in men from sexual minority backgrounds.

## 5 Implications for research

Our findings suggest that structural factors significantly shape experiences of interpersonal conflict among emerging adult men from sexual minority backgrounds. Future research should actively incorporate mixed methods to deepen the understanding of these complex experiences and how they intersect with sexual health. Strengthening collaborations with community-serving organizations and support networks is vital to prioritize the perspectives and lived experiences of men from sexual minority backgrounds. Establishing a Community Advisory Board (CAB) can play a crucial role in this effort, as it would integrate community insights to develop targeted interventions that amplify community strengths and assets related to sexual health. By engaging community members in the research process, the CAB can help identify culturally relevant strategies that promote safer sexual practices, especially for individuals facing housing instability who may be at risk for transactional relationships. This approach not only elevates the voices of community members but also fosters an environment where individuals can access resources and education that support healthier decision-making in their relationships. Longitudinal mixed-methods studies are essential for developing community-informed interventions that not only improve health outcomes but also enhance sexual health literacy among this population. By focusing on building capacity within the community and providing the necessary resources to navigate their circumstances safely, we can work towards mitigating the risks associated with unmet basic needs, such as housing.

## 6 Implications for practice

Healthcare providers, social workers, public health practitioners, and community partners must actively engage with the various factors influencing interpersonal conflict experiences among their clients. By recognizing the interplay of contextual factors, professionals can tailor services to more effectively address the challenges faced by individuals. For instance, incorporating routine assessments of housing stability, employment challenges, and physical safety concerns during clinical visits can facilitate timely referrals to crucial services, such as housing assistance and mental health support. Moreover, healthcare practitioners should be proactive in providing essential resources, such as STI prevention barriers, during these visits. This ensures that individuals are well-equipped to make safer choices if they chose to engage in transactional relationships, particularly in the context of housing instability. Leveraging existing community assets, like local health education programs and peer-led support groups, can enhance resource delivery and empower individuals with vital skills for health advocacy and self-management. The involvement of a Community Advisory Board (CAB) can ensure that clinical practice interventions remain culturally relevant and responsive to community needs, allowing for a more tailored approach in resource provision.

## 7 Implications for policy

Evolving national initiatives require a comprehensive and adaptable public health response that accounts for intersecting social and structural drivers of health. We center evidence-based approaches, including pilot testing, rigorous evaluation, implementation research, and programmatic monitoring, to guide scalable policy decisions. Current gaps in HIV programming infrastructure and limited attention to interpersonal conflict create barriers to care for marginalized groups and warrant focused action. Policy and program planners should support pilot evaluations of interventions that address housing instability, healthcare access, and interpersonal conflict to build an evidence base on effectiveness and scalability. Investments in community health centers and primary care should be paired with evaluated provider training programs that improve cultural responsiveness and care quality. Strengthening workplace protections and enacting policies that reduce structural discrimination should be accompanied by monitoring systems that assess impacts on access and outcomes. Meaningful engagement of community organizations in co-design and evaluation will produce locally relevant evidence and help shape policies that are practical, equitable, and responsive to community needs.

## 8 Limitations

First, generalizability is limited due to the specific ethnicities and identities represented in our sample. Data were collected in English only and may not extend to non-English speakers or cultural groups underrepresented in our recruitment frame. Second, the cross-sectional design of this study restricts the ability to infer causation. While positive associations were identified between contextual factors and experiences of interpersonal conflict, these associations do not imply a causal relationship. Further observational research is necessary to explore the mechanisms linking overlapping identities, societal factors, and interpersonal conflict experiences. Longitudinal studies would provide valuable insights into how the interplay between interpersonal conflict experiences and contextual factors evolves over time. Third, the reliance on self-report may introduce desirability bias, with underreporting of negative experiences or overreporting of positive ones. Fourth, although the intention of the study was to focus on HIV self-testing, potential confounding variables that may influence the relationship between contextual factors and interpersonal conflict, such as mental health status or prior experiences of trauma as well as household income. Fifth, although we used a national recruitment panel to screen over 3,500 individuals and achieve a final sample of 322, selection bias remains a concern. While this approach aims to enhance diversity and representation, self-selection bias may still affect the results, as participants who choose to engage may differ significantly from those who do not, potentially limiting the generalizability of our findings. Despite these limitations, this study has several strengths, including a large sample size that enhanced the statistical power of the findings, extensive geographic recruitment that allowed for a more representative view of the population. Additionally, the wide range of sociodemographic characteristics contributed to a nuanced understanding of the experience’s men from sexual minority backgrounds. The use of validated measures for assessing interpersonal conflict and contextual factors further strengthened the reliability of the data collected.

## 9 Conclusion

This study examined the associations of contextual factors, personal characteristics and experiences of interpersonal conflict among emerging adult, men from sexual minority backgrounds. Age was significantly associated with increased experiences of interpersonal conflict, while Hispanic ethnicity showed a trend toward higher levels of interpersonal conflict, indicating a need for further investigation. Key factors linked to reported interpersonal conflict included lack of health insurance, unemployment, housing instability, and concerns about physical safety. These findings advance the literature by highlighting the complex interplay between identity and social context. Future research should utilize mixed methods and longitudinal designs to identify mechanisms underlying these associations and develop targeted interventions ultimately informing recommendations aimed at promoting health optimization.

## Data Availability

The data analyzed in this study is subject to the following licenses/restrictions: due to the primary focus of the study on HIV risk among sexual minority men, consent was not obtained from participants for data sharing, in compliance with ethical standards and privacy regulations. Requests to access these datasets should be directed to raquel.ramos@yale.edu.
